# Genetic Variation of the Human α-2-Heremans-Schmid Glycoprotein (AHSG) Gene Associated with the Risk of SARS-CoV Infection

**DOI:** 10.1371/journal.pone.0023730

**Published:** 2011-08-17

**Authors:** Xiaohui Zhu, Yan Wang, Hongxing Zhang, Xuan Liu, Ting Chen, Ruifu Yang, Yuling Shi, Wuchun Cao, Ping Li, Qingjun Ma, Yun Zhai, Fuchu He, Gangqiao Zhou, Cheng Cao

**Affiliations:** 1 State Key Laboratory of Pathogen and Bio-Security, Beijing Institute of Biotechnology, Beijing, China; 2 State Key Laboratory of Proteomics, Beijing Proteome Research Center, Beijing Institute of Radiation Medicine, Beijing, China; 3 State Key Laboratory of Pathogen and Bio-Security, Institute of Microbiology and Epidemiology, Beijing, China; 4 General Hospital of Guangzhou Command, PLA, Guangzhou, China; 5 General Hospital of Beijing Command, PLA, Beijing, China; 6 National Engineering Research Center for Protein Drugs, Beijing, China; Kantonal Hospital St. Gallen, Switzerland

## Abstract

Genetic background may play an important role in the process of SARS-CoV infection and SARS development. We found several proteins that could interact with the nucleocapsid protein of the SARS coronavirus (SARS-CoV). α-2-Heremans-Schmid Glycoprotein (AHSG), which is required for macrophage deactivation by endogenous cations, is associated with inflammatory regulation. Cytochrome P450 Family 3A (CYP4F3A) is an *ω*-oxidase that inactivates Leukotriene B4 (LTB4) in human neutrophils and the liver. We investigated the association between the polymorphisms of these two inflammation-associated genes and SARS development. The linkage disequilibrium (LD) maps of these two genes were built with Haploview using data on CHB+JPT (version 2) from the HapMap. A total of ten tag SNPs were selected and genotyped. In the Guangzhou cohort study, after adjusting for age and sex, two *AHSG* SNPs and one *CYP4F3* SNP were found to be associated with SARS susceptibility: rs2248690 (adjusted odds ratio [AOR] 2.42; 95% confidence interval [CI] 1.30-4.51); rs4917 (AOR 1.84; 95% CI 1.02-3.34); and rs3794987 (AOR 2.01; 95% CI 1.10–3.68). To further validate the association, the ten tag SNPs were genotyped in the Beijing cohort. After adjusting for age and sex, only rs2248690 (AOR, 1.63; 95% CI, 1.30–2.04) was found to be associated with SARS susceptibility. The combined analysis of the two studies confirmed tag SNP rs2248690 in *AHSG* as a susceptibility variant (AOR 1.70; 95% CI 1.37–2.09). The statistical analysis of the rs2248690 genotype data among the patients and healthy controls in the HCW cohort, who were all similarly exposed to the SARS virus, also supported the findings. Further, the SNP rs2248690 affected the transcriptional activity of the *AHSG* promoter and thus regulated the AHSG serum level. Therefore, our study has demonstrated that the AA genotype of rs2268690, which leads to a higher AHSG serum concentration, was significantly associated with protection against SARS development.

## Introduction

Severe acute respiratory syndrome (SARS) is an acute respiratory disease resulting from the infection of a previously undescribed coronavirus (SARS-CoV) that spreads through airborne transmission [1]–[3]. Rapid transmission, high infectivity and unpredictable clinical progression with a fatality ratio of approximately 9.6% made SARS a global threat in 2003. However, the pathogenesis of this infectious agent is still not fully understood. Asymptomatic and mildly symptomatic SARS-CoV infections, which represent more than 10% of all SARS-CoV infections, have been reported in many places, including Hong Kong, Taiwan, Guangdong Province of China, and Singapore[4]–[8]. Clinical and laboratory investigations have shown that the host genetic background is an important factor that determines the susceptibility to and pathogenicity of SARS infection. We have demonstrated that genetic haplotypes associated with low serum mannose-binding lectin (*MBL*) are also associated with SARS, and our findings were confirmed by other independent studies [9], [10]. Other susceptible genes, including chemokine (C–C motif) ligand 5 (*CCL5*) [11], interferon gamma (*IFN γ*) [12], 2′–5′ oligoadenylate synthetase 1 (*OAS*) and myxovirus resistance 1 (*MX1*) [13], have also been reported. The involvement of the major histocompatibility complex class I gene (*HLA*) [14]–[16] and the L-SIGN gene (*CLEC4M*) [17]–[19] are still disputed.

Several reports have suggested that the nucleocapsid protein of SARS-CoV, which is expressed at the early stage of viral infection and detectable in the serum [20], is associated with severe pulmonary inflammation by inducing massive pro-inflammatory cytokine production [21]–[23]. In an effort to elucidate this mechanism, human cellular proteins that interact with the nucleocapsid protein were screened by a yeast two-hybrid assay in our laboratory. The translation elongation factor 1α (EEF1A) [24], cytochrome p450 subfamily 4F polypeptide 3 (CYP4F3, Gene ID 4051, GenBank ID NG_007964.1) and alpha-2-HS-glycoprotein (AHSG, Gene ID 197, GenBank ID NG_011436.1) were found to interact with the nucleocapsid protein of SARS-CoV (**Unpublished data**).

AHSG is required for macrophage deactivation by endogenous cations. Low serum AHSG levels may cause the uncontrolled production of proinflammatory cytokines [25], [26]. Mice deficient in AHSG showed hypersensitivity to LPS-induced inflammation [27]. Cytochrome P450 Family 3A (CYP4F3A) is an *ω*-oxidase that inactivates the potent inflammatory factor Leukotriene B4 (LTB4) in human neutrophils and the liver [28]. LTB4 is likely a major determining factor in tissue damage because it can amplify the inflammatory reaction by recruiting leukocytes and inducing their degranulation, which makes the removal of excess LTB4 important for the prevention of immunologic damage [29]. Considering that CYP4F3 and AHSG are involved in innate immunity and inflammatory processes, we investigated whether single-nucleotide polymorphisms (SNPs) in these genes were associated with susceptibility to viral infection.

## Results

### SARS-Cov Nucleocapsid Protein Associates with AHSG

**Figure 1:**
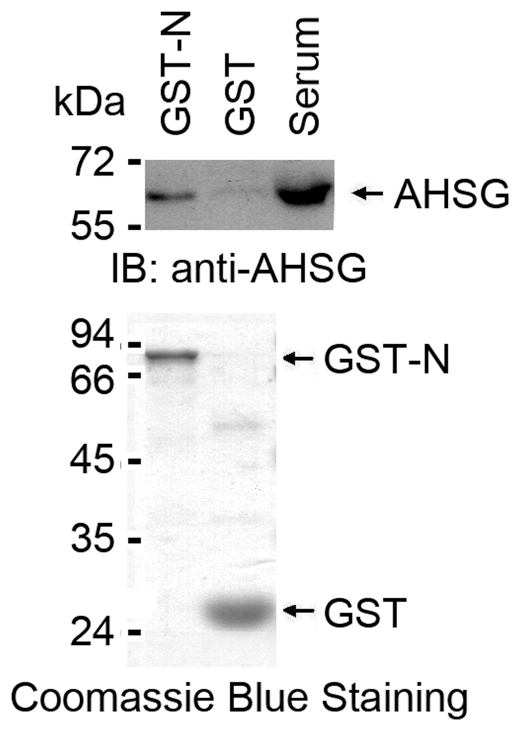
The nucleocapsid protein associates with serum AHSG . Human serum (100 µl) was incubated with agarose beads conjugated to GST-N protein or GST at 4°C for 2 h. The beads were washed three times with PBS supplemented with 0.1% NP40. The bound proteins were subjected to SDS-PAGE followed by immunoblot with anti-AHSG antibody.

We previously noted in a yeast two-hybrid analysis that SARS-Cov nucleocapsid protein associates with CYP4F3A, EEF1A and AHSG (**data not shown**). To substantiate the interaction between SARS-Cov N and AHSG, human serum was incubated with GST or GST-N fusion proteins. The adsorbates were analyzed by immunoblotting with anti-AHSG antibody. It was demonstrated that AHSG binds to GST-N, but not GST (**Figure 1**).

### Study Participants and Demographics

We obtained peripheral blood or serum samples from SARS patients who were diagnosed by WHO standards from two different cities in China. Of the SARS patients, 67 were from the Eighth People's Hospital in southern China City, Guangzhou, and 624 were from Xiaotangshan Hospital (a hospital temporarily set up to fight SARS in Beijing, China) or Ditan Hospital (Beijing, China). All of the SARS samples were collected during the 2003 SARS epidemic. Analysis by ELISA and IFA confirmed all SARS patients to be seropositive for anti-SARS antibodies, and the specificity of the ELISA assay was higher than 99% [20], [30], [31]. For comparison, we obtained two groups of control samples from Guangzhou (192 people) and Beijing (791 people). The Guangzhou control samples were collected during the 2003 SARS epidemic while the Beijing control samples were collected after the epidemic. All samples were collected from people who had never developed SARS. To assess the influence of the exposure, blood samples from health care workers (HCW) that worked in SARS wards in the Second Affiliated Hospital of Senyaxian Medical School in 2003 were obtained. Of these samples, 40 were from HCW who developed SARS during the course of hospital duty and 122 were from health care workers free of the disease. DNA was extracted from all the samples selected. All cases and controls were Han Chinese. We selected enough samples to perform three progressive case-control studies.

Chi-square tests were carried out to evaluate whether the cases and controls of the three studies were matched. We found that the cases and controls from Beijing were well matched for sex and age, while there were more females in the case cohort of the Guangzhou non-HCW population. Most of the HCW population was composed of female nurses between the ages of 16 and 30 (**Table 1**).

**Table 1:** The Demographic Characteristics of the SARS Patients and Controls.

	Guangzhou Non-HCW Population			Beijing Population			HCW Population		
	SARS patients	Controls	*P*	SARS patients	Controls	*P*	SARS patients	Controls	*P*
	(N = 67) No. (%)	(N = 192) No. (%)		(N = 624) No. (%)	(N = 791) No. (%)		(N = 40) No. (%)	(N = 122) No. (%)	
Sex									
Male	29 (43.28)	99 (51.56)	0.24	312 (50.00)	412 (52.09)	0.44	3 (7.50)	12 (9.84)	0.66
Female	38 (56.72)	93 (48.44)		312 (50.00)	379 (47.91)		37 (92.50)	110 (90.16)	
Age, y									
16–30	21 (31.34)	57 (29.69)	0.78	253 (40.54)	339 (42.86)	0.26	29 (72.50)	85 (69.67)	0.96
31–40	23 (34.33)	58 (30.21)		152 (24.36)	163 (20.61)		9 (22.50)	31 (25.41)	
41–50	13 (19.40)	49 (25.52)		131 (21.00)	159 (20.10)		1 (2.50)	4 (3.28)	
>50	10 (14.93)	28 (14.58)		88 (14.10)	130 (16.43)		1 (2.50)	2 (1.64)	

*P* was calculated using the χ^2^ test.

### *AHSG* SNPs and SARS Development

We downloaded the SNP genotype data for CHB+JPT (version 2) from the HapMap database and built a linkage disequilibrium (LD) map of *AHSG* with the Haploview software (version 4.0). The pairwise tagging algorithm implemented in the Tagger program of Haploview (r^2^ threshold was 0.8) was used to select the tag SNPs for assessment. SNPs that were reported to affect the AHSG level or that had been associated with other diseases were also selected. Finally, we chose to genotype rs2248690 (5′-flanking region), rs2077119 (5′-flanking region), rs4917 (exon 6), rs2593813 (intron 1) and rs4918 (exon 7) in the AHSG gene. The genotyping and statistical results are presented in **Table S1** and **Table S2** under the dominant and additive models, respectively. The genotyping and statistical analyses of the Guangzhou non-HCW population revealed that two tag SNPs were associated with SARS. The rs2248690 *TT/AT* genotype was more associated with increased susceptibility to SARS than the *AA* genotype (OR = 2.42; 95% CI, 1.30–4.51; **Table 2** **and Table S1**). The *TT/CT* genotype of rs4917 was associated with an increased possibility of developing clinically apparent SARS (OR = 1.84; 95% CI, 1.02–3.34; **Table 3** **and Table S1**). In the validation study (Beijing population), only the rs2248690 polymorphism was significantly associated with SARS development (relative to the *TT/AT* genotype, 1.63; 95% CI, 1.30–2.04; **Table 2** **and Table S1**). Because the Beijing and Guangzhou sample groups had homogenous demographic and genetic parameters (Han Chinese), a joint analysis was performed. The combined analysis of the two studies under the dominant model is presented in **Table S3**. After combining data from the two cohorts, the *TT/AT* genotype of rs2248690 had a frequency of 27.5% in the control population and a significantly higher frequency of 39.1% in the SARS patients (OR = 1.70; 95% CI, 1.37–2.09; **Table 2** **and Table S3**). After adjusting for sex and age, a non-significant association was observed between rs4917 and SARS susceptibility (OR = 1.22; 95% CI, 1.02–1.54; *P* = 0.08). Because rs4917 and rs2248690 are in high linkage disequilibrium (0.62), the association between rs4917 and SARS can be easily understood. A logistic regression analysis was applied to adjust the linkage between rs4917 and rs2248690. The result showed that rs2248690 was associated with SARS development (OR = 1.58; 95% CI, 1.16–2.17; *p* = 0.004), while rs4917 was not associated with SARS (*p* = 0.54). To remove the influence of the exposure factor to SARS infection and risk, cases and controls from the HCW population were genotyped at rs2248690. Though the *P-*value is higher than 0.05, the distribution of different genotypes between the HCW cases and HCW controls is similar to the distribution between the non-HCW cases and non-HCW controls with a difference of approximately 10%. The statistical analysis of the rs2248690 genotype data from all cases and HCW controls confirmed this finding (OR = 1.68; 95% CI, 1.38–2.06; **Table 4**). We tested the deviation from the Hardy-Weinberg equilibrium in both cases and controls, within each cohort and within the overall study population; we found that there was no statistically significant deviation from equilibrium for any SNP.

**Table 2:** The association results for rs2248690 in two independent samples and the combined sample.

Cohort and Group		Genotype					Allele			
		AA (%)	AT (%)	TT (%)	OR^a^ (95% CI)	*P^a^*	A (%)	T (%)	OR^b^ (95% CI)	*P^b^*
GZ Cohort	Control	145 (75.52)	46 (23.96)	1 (0.52)	2.42 (1.30–4.51)	0.005	336 (87.50)	48 (12.50)	1.93 (1.16–3.22)	0.016
(Non-HCW)	SARS	40 (59.70)	25 (37.31)	2 (2.99)			105 (78.36)	29 (21.64)		
BJ Cohort	Control	545 (71.71)	191 (25.13)	24 (3.16)	1.63 (1.30–2.04)	<0.001	1281 (84.28)	239 (15.72)	1.45 (1.20–1.77)	<0.001
	SARS	369 (60.99)	214 (35.37)	22 (3.64)			952 (78.68)	258 (21.32)		
Combined	Control	690 (72.48)	237 (24.89)	25 (2.63)	1.69 (1.37–2.09)	<0.001	1617 (84.93)	287 (15.07)	1.53 (1.28–1.83)	<0.001
	SARS	409 (60.86)	239 (35.57)	24 (3.57)			1057 (78.65)	287 (21.35)		

CI, confidence interval; BJ, Beijing; GZ, Guangzhou.

a. Case vs. Control. A combined group of the minor-allele homozygotes and heterozygotes was compared with the major-allele homozygotes group ((AT+TT) vs. AA); multivariate unconditional logistic regression was used.

b. Case vs. Control. The frequency of the A allele was compared to the frequency of the T allele; the CMH test was used.

**Table 3:** The association results for rs4917 in two independent samples and the combined sample.

Cohort and Group		Genotype					Allele			
		CC (%)	CT (%)	TT (%)	OR^a^ (95% CI)	*P^a^*	C (%)	T (%)	OR^b^ (95% CI)	*P^b^*
GZ Cohort	Control	98 (56.65)	69 (39.88)	6 (3.47)	1.84 (1.02–3.34)	0.04	265 (76.59)	81 (23.41)	1.49 (0.95–2.33)	0.10
(Non-HCW)	SARS	28 (43.75)	32 (50.00)	4 (6.25)			88 (68.75)	40 (31.25)		
BJ Cohort	Control	391 (53.64)	282 (38.68)	56 (7.68)	1.21 (0.98–1.51)	0.08	1064 (72.98)	394 (27.02)	1.12 (0.95–1.33)	0.18
	SARS	299 (48.07)	281 (45.18)	42 (6.75)			879 (70.66)	365 (29.34)		
Combined	Control	489 (54.21)	351 (38.91)	62 (6.87)	1.22 (1.02–1.54)	0.08	1329 (73.67)	475 (26.33)	1.17 (1.00–1.37)	0.05
	SARS	327 (47.67)	313 (45.63)	46 (6.71)			967 (70.48)	405 (29.52)		

CI, confidence interval; BJ, Beijing; GZ, Guangzhou; HCW, health care workers.

a. Case vs. Control. A combined group of the minor-allele homozygotes and heterozygotes was compared with the major-allele homozygotes group ((CT+TT) vs. CC); multivariate unconditional logistic regression was used.

b. Case vs. Control. The frequency of the C allele was compared to the frequency of the T allele; the CMH test was used.

**Table 4:** Evaluation of the Exposure Factor.

Cohorts compared	Genotype	No. Cases (%)	No. Controls (%)	Crude OR^a^ (95% CI)	*P^a^* Value	Adjusted OR^b^ (95% CI)	*P^b^* Value
HCW Cases vs. HCW Controls	AA	25 (62.50)	88 (72.10)	1[Reference]		1[Reference]	
	TT/AT	15 (37.50)	34 (27.90)	1.55 (0.73–3.30)	0.32	1.54 (0.72–3.28)	0.27
GZ Cases vs. HCW Controls	AA	65 (60.75)	88 (72.13)	1[Reference]		1[Reference]	
	TT/AT	42 (39.25)	34 (27.87)	1.67 (0.96–2.91)	0.09	1.73 (0.94–3.18)	0.08
All Cases vs. HCW Controls	AA	434 (60.96)	88 (72.13)	1[Reference]		1[Reference]	
	TT/AT	278 (39.04)	34 (27.87)	1.66 (1.09–2.53)	0.02	1.58 (1.00–2.47)	0.05
All Cases vs. All controls	AA	434 (60.96)	778 (72.44)	1[Reference]		1[Reference]	
	TT/AT	278 (39.04)	296 (27.56)	1.68 (1.38–2.06)	<0.001	1.68 (1.38–2.06)	<0.001

Abbreviations used: CI, confidence interval; OR, odds ratio; HCW, health care workers; GZ, Guangzhou. All OR and *P* values are from the reference group but against the other category.

a Values calculated using the CMH test.

b Values adjusted for age and sex using the multivariate unconditional logistic regression.

Based on these results, we conclude that the *TT/AT* genotype of rs2248690 is associated with the increased likelihood of developing SARS, while the *AA* genotype is associated with protection against SARS.

### rs2248690 is associated with AHSG serum concentration

AHSG is a serum protein, and it has been reported that there is an association between *AHSG* polymorphisms (rs4917 and rs4918) and AHSG serum concentration levels [32], [33]. However, no convincing multivariate analysis has been performed to identify the most associated variants. To uncover potential functional changes associated with the *AHSG* rs2248690 polymorphism, 192 healthy subjects from Beijing were genotyped, and their AHSG serum concentrations were determined. As expected, there was an association between the rs2248690 genotype and the AHSG serum concentrations (**Table 5**). The order of the average AHSG serum concentrations was as follows: *−799AA* >*−799AT* >*−799TT*. Consistently, rs4917 and rs4918 were associated with different serum levels of AHSG. Furthermore, a stepwise regression analysis clearly showed that rs2248690 was an independent contributor to the variability in AHSG serum concentration (**Table 5**). These results show that LD with rs2248690 causes the association of rs4917 with AHSG serum concentration.

**Table 5:** Variants Affecting AHSG Serum Levels.

Variables	*β*	*T*	*P* a
rs2248690	−0.737	−14.336	0.0001
rs4917	0.014	0.209	0.84
rs4918	−0.011	−0.162	0.87
Sex	−0.057	−1.11	0.27
Age	0.002	0.046	0.96
**rs2248690 Genotype**	**N (%)**	**AHSG concentration (mean ± S.E.M) µg/ml**	***P*** **(ANOVA)**
AA	142 (74.0%)	583±33	
AT	45 (23.4%)	541±25	<0.001
TT	5 (2.6%)	492±28	

a Stepwise multivariate regression analysis.

**Figure 2:**
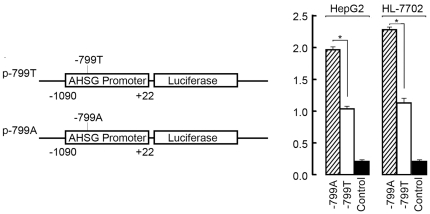
The rs2248690(−*799A/T*) polymorphism regulates AHSG promoter activity. Left, a schematic of two reporter gene constructs. Right, luciferase expression of the constructs in different cells. Each value represents the mean ± S.D. of five independent experiments that were performed in triplicate. *, P<0.05.

Because rs2248690 is located in the promoter region of the AHSG gene (−799 bp upstream of the transcription start site), various alleles of this SNP may yield phenotypic differences in *AHSG* transcription levels. It has been reported that the A allele of rs2248690 has a reduced binding affinity for transcription factor AP1 [34], which is a repressor of AHSG expression [35], [36]. We observed allele-associated differences in the *AHSG* promoter's transcriptional activity in two different cell lines using a luciferase assay. The sequence that contained −*799T*, which is overrepresented in SARS patients, showed significantly lower promoter activity (**Figure 2**).

### *CYP4F3* SNPs and SARS Susceptibility

We also built an LD map of *CYP4F3* and selected five tag SNPs for assessment (**Table S1, Table S2 and Table S3**). In the non-HCW Guangzhou population, the rs3794987 *GG/AG* genotype was associated with an increased susceptibility to SARS (OR = 2.01; 95% CI, 1.10–3.68). However, the results of this association were not replicated in the Beijing population. The combined analysis of the two studies does not show any association of the *CYP4F3* SNPs analyzed with SARS susceptibility.

## Discussion

After the interaction between AHSG and the SARS-CoV nucleocapsid protein was identified and validated, we chose *AHSG* as a candidate gene in subsequent case-control analyses. We found an association between one SNP in *AHSG* (rs2248690) and the development of SARS in two separate case-control studies as well as in the combined analysis of both studies after adjusting for age and sex. Considering the exposure factor, the intercomparison of the HCW-controls and the other cases validated the association we observed between rs2248690 and SARS disease because the HCW-controls, who had worked in SARS wards, were exposed to SARS-CoV at least as much as the other cases. Additionally, this polymorphism, which was located in the promoter of *AHSG*, affects AHSG serum levels by altering the transcriptional activity. The genotype that conferred protection against SARS, rs2248690 *AA*, is associated with higher AHSG serum levels.

Our case-control studies included 1833 individuals and revealed a significant association between rs2248690 and the SARS disease. More specifically, individuals with the *AA* genotype have a 41% lower risk of developing SARS than those with the *TT/AT* genotype. The rs2248690 SNP is located in the 5′ flanking region of *AHSG* at position −799 within the promoter region. We have shown that this variant affects AHSG serum levels by altering transcriptional activity. Other SNPs in the *AHSG*, such as rs4917 and rs4918, have also been shown to be associated with AHSG serum levels and diseases affected by AHSG serum levels [33]. In this study, we demonstrated that rs2248690 is the dominant factor affecting AHSG concentration.

AHSG is a circulating negative inflammatory acute-phase glycoprotein. The pathophysiological sequence of infection is mediated by macrophage-derived pro-inflammatory cytokines. The current literature suggests that the availability of AHSG to macrophages is critical in regulating the innate immune response in tissue injury and infection because AHSG is required for macrophage deactivation by endogenous cations [37]. Decreased levels of human fetuin have been observed in patients with acute lymphocytic Hodgkin's and non-Hodgkin's lymphomas, rheumatoid arthritis, acute alcoholic hepatitis and trauma [28], [38]–[41]. Under conditions where macrophage-associated fetuin levels are decreased, macrophage deactivation by endogenous counter-regulators may be impaired, leading to the uncontrolled overproduction of pro-inflammatory cytokines [29], [38]. A recent work showed that mice deficient in AHSG are hypersensitive to LPS-induced inflammation [27]. Because the development of SARS is always associated with a severe inflammatory reaction, low AHSG serum levels could result in severe symptoms after a SARS-CoV infection. Considering that there might be many asymptomatic SARS-CoV infections in the control group, it is plausible that relatively higher serum concentrations of AHSG can orchestrate an unimpaired counter-regulation between macrophage deactivation and endogenous pro-inflammatory cytokines. Therefore, viruses can be cleared without severe inflammation and obvious symptoms. However, we observed that AHSG interacts with the SARS-CoV nucleocapsid protein (**Figure 1**), which may induce massive pro-inflammatory cytokine production upon infection [21]. AHSG may neutralize the pro-inflammatory effect of the SARS nucleocapsid protein because AHSG is so abundant in the serum (0.2–0.55 mg/ml). Considering that AHSG is a circulating glycoprotein synthesized in human liver tissue and the nucleocapsid protein is “wrapped” by membrane proteins in the SARS-CoV virion, interactions between AHSG and the nucleocapsid protein are less likely to affect the viral infectivity and replication of the SARS-CoV.

To our knowledge, this is the first study reporting an association of *AHSG* polymorphisms with SARS. Individuals with polymorphisms that contribute to high concentrations of AHSG may be less likely to develop SARS. These findings improve our understanding of SARS pathogenesis and provide a genetic basis for SARS prognosis. Future studies should further investigate the mechanisms of AHSG-based resistance to SARS and other infectious diseases.

## Materials and Methods

### Ethics Statement

Written, informed consent was obtained from all of the participants or their guardians; genetic analysis was covered in the consent. This study was performed with the approval of the Ethics Committees of all the hospitals from which samples were obtained and it was also approved by the the Ethics Committees of Chinese National Human Genome Center, Beijing Institute of Biotechnology and Beijing Proteome Research Center. The study was conducted according to the principles of the Declaration of Helsinki.

### Study Participants

The first case-control study was performed to evaluate potential associations between *AHSG* and *CYP4F3* polymorphisms with SARS. The second case-control study was performed to confirm the findings from the first study. The HCW case-control study was used to evaluate the influence of the exposure rate and to reconfirm the findings of positively associated SNPs. The association of the *AHSG* genotype and AHSG serum concentrations was evaluated in 192 healthy Beijing Han Chinese.

### Case-Control Study 1

Serum samples from 67 SARS patients diagnosed by WHO standards were collected from the Eighth People's Hospital of Guangzhou in 2003. Analysis by enzyme-linked immunosorbent assay (ELISA) and immunofluorescent assay (IFA) confirmed that all of the SARS patients were seropositive for SARS antibodies. The specificity of the ELISA assay was higher than 99% [20], [30], [31]. Serum samples from 192 age-, sex-, and ethnicity-matched controls were collected from healthy people in Guangzhou during the same period. All cases and controls were Han Chinese.

### Case-Control Study 2

Blood samples from 624 patients diagnosed by WHO standards were collected from Xiaotangshan Hospital (a hospital temporarily set up to fight SARS in Beijing, China) and Ditan Hospital (Beijing, China) during the SARS epidemic in 2003. All SARS patients selected were confirmed to be seropositive by both an enzyme-linked immunosorbent assay (ELISA) and an immunofluorescent assay (IFA) [20], [30], [31]. Information regarding sex, ethnic status, and age at SARS diagnosis was recorded. A total of 791 age-, sex-, and ethnicity-matched adults were recruited as control subjects after the 2003 SARS epidemic, and none of these control subjects ever developed SARS (**Table 1**). Of these samples, 352 infected patients (from Xiaotangshan Hospital) and 410 controls were from the BPRC (Beijing Proteome Research Center) of China and were previously employed in a study that showed no association between *CLEC4M* homozygosity and protection against SARS coronavirus [18]. All cases and controls were Han Chinese. The remaining 272 samples of SARS patients were collected from the Ditan Hospital.

### HCW Population Case-Control Study

Blood samples were obtained from 40 health care workers that developed SARS during the course of hospital duty and 122 health care workers who had worked in SARS wards but remained free of the disease. All of these HCW workers worked in SARS wards in the Second Affiliated Hospital of Senyaxian Medical School in 2003. Information regarding sex and age was recorded. The HCW cases and controls were Han Chinese.

### rs2248690 Genotype and AHSG Serum Concentration study

Blood samples from 192 healthy Beijing Chinese were collected from the 307 Hospital of PLA (People's Liberation Army) in Beijing, and sera were collected. Serum AHSG levels were assessed by ELISA, and the rs2248690 loci were genotyped as described below.

### DNA Isolation

Peripheral blood samples were obtained from Guangzhou HCW and Beijing cohorts. A total of 200 µl of each blood sample was mixed and incubated with 1×RBC lysis buffer (1.5 M ammonium chloride, 100 mM potassium bicarbonate and 10 mM EDTA). Subsequently, the lymphocytes were obtained by centrifugation. Serum samples were obtained from the Guangzhou non-HCW cases and controls. Genomic DNA from all the peripheral blood leukocytes and plasma samples was extracted using the QIAamp DNA Blood Mini Kit (Qiagen, Valencia, CA) and stored at −20°C. The resulting DNA was quantified using a standard spectrophotometric reading on a DU 650 spectrophotometer (Beckman Coulter,Fullerton, CA). Each sample was diluted to a final concentration of 10 ng/ml.

### Selection of SNPs

For the association study, we downloaded the SNP genotype data for CHB+JPT (version 2) from the HapMap database and built LD maps of these two genes with the Haploview software. A pairwise tagging algorithm implemented in the Tagger program of Haploview (with an r^2^ threshold of 0.8) was used to select the tag SNPs. SNPs that were reported to affect AHSG/CYP4F3 levels or had been associated with other diseases were also selected. Finally, we chose to genotype rs2248690 (5′-flanking region), rs2077119 (5′-flanking region), rs4917 (exon 6), rs2593813 (intron 1), and rs4918 (exon 7) in the AHSG gene. The *CYP4F3* gene has more than 30 SNPs in three linkage disequilibrium blocks. We chose rs3794987 (5′-flanking region), rs1159776 (5′-flanking region), rs4646519 (intron 11), and rs1290625 (intron 2) for genotyping.

### Genotyping

Genotyping was completed using the SNPstream ultra-high throughput genotyping system (Beckman Coulter, Fullerton, CA) according to the manufacturer's instructions. Briefly, the method combines solution-phase multiplex single nucleotide extension (SNE) with a solid-phase sorting of labeled SNE primers by hybridization to a chip that contains 384 4×4 arrays of 12 oligonucleotide tags and 4 oligonucleotides for positive and negative controls. Each SNE primer contained 1 of the 20 oligonucleotide tags at its 5′ end, and the SNE reactions were performed in 12-plex. The microarray plate was imaged using a SNP scope reader (Beckman Coulter, Fullerton, CA). The two-color system allowed the detection of the SNP by comparing the signals from the two fluorescent dyes. The image signals were subsequently transferred to genotyping software that translated the images of the arrays into genotype calls. The error rate (0–1.2%) was determined by DNA sequencing (3730, Applied Biosystem Inc) for 10% of the SNPs examined in the present study.

### Statistical Analysis

Chi-square tests were used to compare the genotypic frequencies and demographic distributions between the cases and controls. The exact test was applied to evaluate whether the population was in Hardy–Weinberg equilibrium by the Markov chain method; P-values less than 0.05 were considered statistically significant. The tag SNPs were chosen on a pairwise basis, and the linkage disequilibrium (LD) calculation was performed on a confidence interval (CI) basis using the Haploview 4.0 software.

Fisher's exact test and multivariate unconditional logistic regression were used to estimate the crude and adjusted odds ratios for each sex and age category. The odds ratio and a 95% CI were used to measure the strength of association in the genetic risk association study. The additive and dominant models were used for this analysis. In the additive model, each SNP was genotyped and separated into three categories with the homozygous major allele genotype chosen as the reference group. For the dominant model, each SNP was modeled as a dichotomous variable with the homozygous major allele genotype as the reference group and the other two genotypes combined into one category.

ANOVA and stepwise multivariate regression analyses were performed to identify the SNPs associated with AHSG serum levels. These statistical analyses were performed using SAS (version 8.0).

### Serum AHSG assay

Serum AHSG concentrations were measured in 192 subjects by ELISA. Briefly, the sera were diluted 10,000-fold and pipetted into the wells of a microtiter plate whose surfaces were coated with polyclonal anti-human AHSG antibodies (R&D). After incubation, horseradish peroxidase (HRP) -conjugated polyclonal anti-human AHSG antibodies were added to the wells. Following the incubation and washing steps, the absorbance was measured using an automated plate reader at 450 nm. The concentrations of the diluted sera were read from the standard curve of human AHSG, and the concentrations of the experimental samples were calculated. All samples were measured in parallel and in duplicate. The intra- and inter-assay coefficients of variations were below 10%. The statistical analyses were done using ANOVA.

### Promoter assays

Two reporter plasmids encompassing −1091 to +22 bp of the human AHSG promoter were generated by PCR from two genomic DNA samples with either −799A OR -799T, using the primers P1: 5′-cgcagatctGGTCTCCATGAGAGGGCTTC-3′ and P2: 5′-cgcaagcttCAGGCGTGCAGGTGGTTG-3′, respectively. The PCR product was digested with BglII and HindIII and ligated into a pGL3-basic promoter-probing vector (Promega) containing the firefly luciferase gene as a reporter. The constructs were sequenced to ensure correct cloning.

HepG2 and HL7702 cells were seeded at 1×10^5^ cells per well, respectively, in 12-well plates and transfected with pGL3-basic (a promoterless control) or pGL3-basic containing a particular allele of the *AHSG* promoter. The pRL-SV40 plasmid (Promega) was cotransfected as a normalizing control. All transfections were carried out in triplicate. At 24 h post-incubation, the cells were collected and analyzed for luciferase activity using the Dual-Luciferase Reporter Assay System (Promega). The data shown represent three independent experiments performed in triplicate. The statistical analyses were performed using the Student's *t*-test.

### Protein-binding assays

For the GST pull-down experiments, human serum were incubated for 2 h at 4°C with 5 µg of purified GST or GST fusion proteins bound to glutathione beads (GE Healthcare, Little Chalfont, Buckinghamshire, UK). The adsorbates were washed with lysis buffer and subjected to SDS-PAGE and immunoblot analysis. Purified AHSG protein was included as a loading control.

## Supporting Information

Table S1 **The Dominant Model of Crude and Adjusted Odd Ratios (ORs) by** ***AHSG*** **and** ***CYP4F3*** **Single-Nucleotide Polymorphism (SNP) Genotypes.**
(DOC)

Table S2 **The Additive model of Crude and Adjusted Odd Ratios (ORs) by** ***AHSG*** **and** ***CYP4F3*** **Single-Nucleotide Polymorphism (SNP) Genotypes.**
(DOC)

Table S3 **Combinatorial Analysis Under the Dominant Model.**
(DOC)

## References

[1] Peiris JS, Lai ST, Poon LL, Guan Y, Yam LY (2003). Coronavirus as a possible cause of severe acute respiratory
syndrome.. Lancet.

[2] Ksiazek TG, Erdman D, Goldsmith CS, Zaki SR, Peret T (2003). A novel coronavirus associated with severe acute respiratory
syndrome.. N Engl J Med.

[3] Drosten C, Gunther S, Preiser W, van der Werf S, Brodt HR (2003). Identification of a novel coronavirus in patients with severe
acute respiratory syndrome.. N Engl J Med.

[4] Woo PC, Lau SK, Tsoi HW, Chan KH, Wong BH (2004). Relative rates of non-pneumonic SARS coronavirus infection and
SARS coronavirus pneumonia.. Lancet.

[5] Chang WT, Kao CL, Chung MY, Chen SC, Lin SJ (2004). SARS exposure and emergency department workers.. Emerg Infect Dis.

[6] Li G, Zhao Z, Chen L, Zhou Y (2003). Mild severe acute respiratory syndrome.. Emerg Infect Dis.

[7] Ho KY, Singh KS, Habib AG, Ong BK, Lim TK (2004). Mild illness associated with severe acute respiratory syndrome
coronavirus infection: lessons from a prospective seroepidemiologic study of
health-care workers in a teaching hospital in Singapore.. J Infect Dis.

[8] Wilder-Smith A, Teleman MD, Heng BH, Earnest A, Ling AE (2005). Asymptomatic SARS coronavirus infection among healthcare workers,
Singapore.. Emerg Infect Dis.

[9] Zhang H, Zhou G, Zhi L, Yang H, Zhai Y (2005). Association between mannose-binding lectin gene polymorphisms and
susceptibility to severe acute respiratory syndrome coronavirus
infection.. J Infect Dis.

[10] Ip WK, Chan KH, Law HK, Tso GH, Kong EK (2005). Mannose-bingding lectin in severe acute respiratory syndrome
coronavirus infection.. J Infect Dis.

[11] Ng MW, Zhou G, Chong WP, Lee LW, Law HK (2007). The association of RANTES polymorphism with severe acute
respiratory syndrome in Hong Kong and Beijing Chinese.. BMC Infect Dis.

[12] Chong WP, Ip WK, Tso GH, Ng MW, Wong WH (2006). The interferon gama gene polymorphism +874 A/T is associated
with severe acute respiratory syndrome.. BMC Infect Dis.

[13] He J, Feng D, de Vlas SJ, Wang H, Fontanet A (2006). Association of SARS susceptibility with single nucleic acid
polymorphisms of OAS1 and MxA genes: a case-control study.. BMC Infect Dis.

[14] Lin M, Tseng HK, Trejaut JA, Lee HL, Loo JH (2003). Association of HLA class I with severe acute respiratory syndrome
coronavirus infection.. BMC Med Genet.

[15] Ng MH, Lau KM, Li L, Cheng SH, Chan WY (2004). Association of human-leukocyte-antigen class I (B*0703) and
class II (DRB1*0301) genotypes with susceptibility and resistance to the
development of severe acute respiratory syndrome.. J Infect Dis.

[16] Xiong P, Zeng X, Song MS, Jia SW, Zhong MH (2008). Lack of association between HLA-A, -B and -DRB1 alleles and the
development of SARS: a cohort of 95 SARS-recovered individuals in a
population of Guangdong, southern China.. Int J Immunogenet.

[17] Chan VS, Chan KY, Chen Y, Poon LL, Cheung AN (2006). Homozygous L-SIGN (CLEC4M) plays a protective role in SARS
coronavirus infection.. Nat Genet.

[18] Zhi L, Zhou G, Zhang H, Zhai Y, Yang H (2007). Lack of support for an association between CLEC4M homozygosity
and protection against SARS coronavirus infection.. Nat Genet.

[19] Tang NL, Chan PK, Hui DS, To KF, Zhang W (2007). Lack of support for an association between CLEC4M homozygosity
and protection against SARS coronavirus infection.. Nat Genet.

[20] Che XY, Hao W, Wang Y, Di B, Yin K (2004). Nucleocapsid protein as early diagnostic marker for
SARS.. Emerg Infect Dis.

[21] Yasui F, Kai C, Kitabatake M, Inoue S, Yoneda M (2008). Prior immunization with severe acute respiratory syndrome
(SARS)-associated coronavirus (SARS-CoV) nucleocapsid protein causes severe
pneumonia in mice infected with SARS-CoV.. J Immunol.

[22] Zhao J, Huang Q, Wang W, Zhang Y, Lv P (2007). Identification and characterization of dominant helper T-cell
epitopes in the nucleocapsid protein of severe acute respiratory syndrome
coronavirus.. J Virol.

[23] Zhu MS, Pan Y, Chen HQ, Shen Y, Wang XC (2004). Induction of SARS-nucleoprotein-specific immune response by use
of DNA vaccine.. Immunol Lett.

[24] Zhou B, Liu J, Wang Q, Liu X, Li X (2008). The nucleocapsid protein of severe acute respiratory syndrome
coronavirus inhibits cell cytokinesis and proliferation by interacting with
translation elongation factor 1alpha.. J Virol.

[25] Lebreton JP, Joisel F, Raoult JP, Lannuzel B, Rogez JP (1979). Serum concentration of human alpha 2 HS glycoprotein during the
inflammatory process: evidence that alpha 2 HS glycoprotein is a negative
acute-phase reactant.. J Clin Invest.

[26] Ombrellino M, Wang H, Yang H, Zhang M, Vishnubhakat J (2001). Fetuin, a negative acute phase protein, attenuates TNF synthesis
and the innate inflammatory response to carrageenan.. Shock.

[27] Li W, Zhu S, Li J, Huang Y, Zhou R (2011). A hepatic protein, fetuin-A, occupies a protective role in lethal
systemic inflammation.. PLoS One.

[28] Murphy RC, Gijon MA (2007). Biosynthesis and metabolism of leukotrienes.. Biochem J.

[29] Kannan S (2002). Amplification of extracellular nucleotide-induced leukocyte(s)
degranulation by contingent autocrine and paracrine mode of
leukotriene-mediated chemokine receptor activation.. Med Hypotheses.

[30] Liu X, Shi Y, Li P, Li L, Yi Y (2004). Profile of antibodies to the nucleocapsid protein of the severe
acute respiratory syndrome (SARS)-associated coronavirus in probable SARS
patients.. Clin Diagn Lab Immunol.

[31] Shi Y, Yi Y, Li P, Kuang T, Li L (2003). Diagnosis of severe acute respiratory syndrome (SARS) by
detection of SARS coronavirus nucleocapsid antibodies in an
antigen-capturing enzyme-linked immunosorbent assay.. J Clin Microbiol.

[32] Osawa M, Umetsu K, Ohki T, Nagasawa T, Suzuki T (1997). Molecular evidence for human alpha 2-HS glycoprotein (AHSG)
polymorphism.. Human Genetics.

[33] Osawa M, Tian W, Horiuchi H, Kaneko M, Umetsu K (2005). Association of alpha2-HS glycoprotein (AHSG, fetuin-A)
polymorphism with AHSG and phosphate serum levels.. Human Genetics.

[34] Inoue M, Takata H, Ikeda Y, Suehiro T, Inada S (2008). A promoter polymorphism of the alpha2-HS glycoprotein gene is
associated with its transcriptional activity.. Diabetes Res Clin Pract.

[35] Bushel P, Kim JH, Chang W, Catino JJ, Ruley HE (1995). Two serum response elements mediate transcriptional repression of
human smooth muscle alpha-actin promoter in ras-transformed
cells.. Oncogene.

[36] Schreiber M, Kolbus A, Piu F, Szabowski A, Möhle-Steinlein U (1999). Control of cell cycle progression by c-Jun is p53
dependent.. Genes & development.

[37] Wang H, Zhang M, Bianchi M, Sherry B, Sama A (1998). Fetuin (alpha2-HS-glycoprotein) opsonizes cationic
macrophagedeactivating molecules.. Proc Natl Acad Sci U S A.

[38] Kalabay L, Cseh K, Benedek S, Fekete S, Masszi T (1991). Serum alpha 2-HS glycoprotein concentration in patients with
hematological malignancies. A follow-up study.. Ann Hematol.

[39] Sato H, Kazama JJ, Wada Y, Kuroda T, Narita I (2007). Decreased levels of circulating alpha2-Heremans-Schmid
glycoprotein/Fetuin-A (AHSG) in patients with rheumatoid
arthritis.. Intern Med.

[40] Jezequel M, Seta N, Corbic M, Feger J, Durand G (1988). Modifications of concanavalin A patterns of A1-acid-glyco-protein
and α2-HS-glycoprotein in alcoholic liver disease.. Clin Chim Acta.

[41] Kalabay L, Cseh K, Pozsonyi T, Jakab L, Jakab L (1992). Diagnostic value of the determination of serum
α2-HS-glycoprotein concentration.. Orv Hetil.

