# Supplementary material for: Genetic Variation of the Human α-2-Heremans-Schmid Glycoprotein (AHSG) Gene Associated with the Risk of SARS-CoV Infection
Source: PLoS One. 2011 Aug 17;6(8):e23730. doi: 10.1371/journal.pone.0023730 (PMC3163911; doi:10.1371/journal.pone.0023730)
Supplement: Table S1 — The Dominant Model of Crude and Adjusted Odd Ratios (ORs) by AHSG and CYP4F3 Single-Nucleotide Polymorphism (SNP) Genotypes. (DOC) [file pone.0023730.s001.doc]

**Table S1** Dominant Model of Crude and Adjusted Odd Ratios (ORs) by *AHSG* and *CYP4F3* Single-Nucleotide Polymorphism (SNP) Genotypes

|  | | | | | | | | | | | |
| --- | --- | --- | --- | --- | --- | --- | --- | --- | --- | --- | --- |
|  |  | **Case-Control Study 1 (Guangzhou Non-HCW Population)** | | | | | **Case-Control Study 2 (Beijing Population)** | | | | |
| **SNP** | **Genotype** | **No. of Cases/ No. of Controls** | **Crude OR (95% CI)** | ***P* Value** | **Adjusted OR (95% CI)^a^** | ***P* Value** | **No. of Cases/ No. of Controls** | **Crude OR (95% CI)** | ***P* Value** | **Adjusted OR (95% CI)^a^** | ***P* Value** |
| ***AHSG*** | | | | | | | | | | | |
| rs2248690 | AA | 40/145 | 1 [Reference] |  | 1 [Reference] |  | 369/545 | 1 [Reference] |  | 1 [Reference] |  |
|  | TT/AT | 27/47 | 2.08 (1.16-3.75) | **.02** | 2.42 (1.30-4.51) | **.005** | 236/215 | 1.62 (1.29-2.03) | **<.001** | 1.63 (1.30-2.04) | **<.001** |
| rs4917 | CC | 28/98 | 1 [Reference] |  | 1 [Reference] |  | 299/391 | 1 [Reference] |  | 1 [Reference] |  |
|  | TT/CT | 36/75 | 1.68 (0.94-3.00) | .08 | 1.84 (1.02-3.34) | **.04** | 323/338 | 1.25 (1.01-1.55) | **.04** | 1.21 (0.98-1.51) | .08 |
| rs2077119 | CC | 25/81 | 1 [Reference] |  | 1 [Reference] |  | 193/199 | 1 [Reference] |  | 1 [Reference] |  |
|  | AA/AC | 42/111 | 1.23 (0.69-2.17) | .56 | 1.48 (0.81-2.71) | .21 | 356/354 | 1.04 (0.81-1.33) | .80 | 1.13 (0.88-1.46) | .35 |
| rs2593813 | TT | 33/93 | 1 [Reference] |  | 1 [Reference] |  | 281/362 | 1 [Reference] |  | 1 [Reference] |  |
|  | CC/CT | 26/86 | 0.85 (0.47-1.54) | .65 | 0.93 (0.51-1.71) | .81 | 291/340 | 1.10 (0.88-1.38) | .40 | 1.26 (0.99-1.59) | .06 |
| rs4918 | CC | 36/111 | 1 [Reference] |  | 1 [Reference] |  | 302/430 | 1 [Reference] |  | 1 [Reference] |  |
|  | GG/CG | 31/81 | 1.18 (0.67-2.06) | .57 | 1.27 (0.71-2.28) | .42 | 278/355 | 1.12 (0.90-1.38) | .32 | 1.17 (0.93-1.48) | .19 |
| ***CYP4F3*** | | | | | | | | | | | |
| rs3794987 | AA | 35/120 | 1 [Reference] |  | 1 [Reference] |  | 201/200 | 1 [Reference] |  | 1 [Reference] |  |
|  | GG/AG | 32/66 | 1.66 (0.94-2.93) | .08 | 2.01 (1.10-3.68) | **.02** | 109/139 | 0.78 (0.57-1.07) | .15 | 1.35 (0.94-1.95) | .11 |
| rs1159776 | AA | 42/126 | 1 [Reference] |  | 1 [Reference] |  | 432/518 | 1 [Reference] |  | 1 [Reference] |  |
|  | GG/AG | 20/66 | 0.91 (0.49-1.67) | .88 | 1.06 (0.55-2.03) | .86 | 174/250 | 0.83 (0.66-1.05) | .14 | 0.84 (0.67-1.07) | .15 |
| rs4646519 | TT | 23/68 | 1 [Reference] |  | 1 [Reference] |  | 142/129 | 1 [Reference] |  | 1 [Reference] |  |
|  | CC/CT | 37/112 | 0.98 (0.54-1.78) | 1.00 | 0.76 (0.40-1.44) | .41 | 184/189 | 0.88 (0.65-1.21) | .47 | 0.91 (0.66-1.26) | .57 |
| rs4343407 | CC | 36/88 | 1 [Reference] |  | 1 [Reference] |  | 137/198 | 1 [Reference] |  | 1 [Reference] |  |
|  | TT/CT | 31/104 | 0.73 (0.42-1.27) | .32 | 0.86 (0.48-1.55) | .62 | 132/220 | 0.87 (0.64-1.18) | .39 | 0.89 (0.65-1.22) | .46 |
| rs2683038 | GG | 46/119 | 1 [Reference] |  | 1 [Reference] |  | 183/273 | 1 [Reference] |  | 1 [Reference] |  |
|  | CC/CG | 21/73 | 0.74 (0.41-1.35) | .38 | 0.56 (0.30-1.05) | .07 | 75/138 | 0.81 (0.58-1.14) | .23 | 0.71 (0.49-1.01) | .06 |

Abbreviation: CI, confidence interval; OR, odds ratio; SNP, nucleotide polymorphism. All OR and P value are of the reference group but against the other category.

a Values adjusted for age, sex.
