# Supplementary material for: Genetic Variation of the Human α-2-Heremans-Schmid Glycoprotein (AHSG) Gene Associated with the Risk of SARS-CoV Infection
Source: PLoS One. 2011 Aug 17;6(8):e23730. doi: 10.1371/journal.pone.0023730 (PMC3163911; doi:10.1371/journal.pone.0023730)
Supplement: Table S2 — The Additive model of Crude and Adjusted Odd Ratios (ORs) by AHSG and CYP4F3 Single-Nucleotide Polymorphism (SNP) Genotypes. (DOC) [file pone.0023730.s002.doc]

**Table S2** Additive model of Crude and Adjusted Odd Ratios (ORs) by *AHSG* and *CYP4F3* Single-Nucleotide Polymorphism (SNP) Genotypes

|  | | | | | | | | | | | |
| --- | --- | --- | --- | --- | --- | --- | --- | --- | --- | --- | --- |
|  | | **Case-Control Study 1 ( Guangzhou Non-HCW Population )** | | | | | **Case-Control Study 2 (Beijing Population)** | | | | |
| **SNP** | **Genotype** | **No. of Cases/ No. of Controls** | **Crude OR (95% CI)** | ***P* Value** | **Adjusted OR (95% CI)^a^** | ***P* Value** | **No. of Cases/ No. of Controls** | **Crude OR (95% CI)** | ***P* Value** | **Adjusted OR (95% CI)^a^** | ***P* Value** |
| ***AHSG*** | | | | | | | | | | | |
| rs2248690 | AA | 40/145 | 1[Reference] |  | 1[Reference] |  | 369/545 | 1[Reference] |  | 1[Reference] |  |
|  | AT | 25/46 | 1.97 (1.08-3.59) | .**04** | 2.28 (1.21-4.28) | **.01** | 214/191 | 1.65 (1.31-2.09) | **<.001** | 1.66 (1.31-2.10) | **<.001** |
|  | TT | 2/1 | 7.25 (0.64-82.01) | .13 | 7.50 (0.81-69.69) | .08 | 22/24 | 1.35 (0.75-2.45) | .36 | 1.37 (0.76-2.49) | .30 |
| rs4917 | CC | 28/98 | 1[Reference] |  | 1[Reference] |  | 299/391 | 1[Reference] |  | 1[Reference] |  |
|  | CT | 32/69 | 1.62 (0.90-2.94) | .13 | 1.86 (1.01-3.45) | .05 | 281/282 | 1.30 (1.04-1.63) | **.02** | 1.24 (0.99-1.56) | .06 |
|  | TT | 4/6 | 2.33 (0.62-8.85) | .24 | 2.64 (0.69-10.11) | .16 | 42/56 | 0.98 (0.64-1.50) | 1.00 | 1.02 (0.66-1.56) | .94 |
| rs2077119 | CC | 25/81 | 1[Reference] |  | 1[Reference] |  | 193/199 | 1[Reference] |  | 1[Reference] |  |
|  | AC | 34/83 | 1.33 (0.73-2.42) | .37 | 1.64 (0.87-3.10) | .12 | 282/274 | 1.06 (0.82-1.37) | .69 | 1.16 (0.89-1.52) | .27 |
|  | AA | 8/28 | 0.93 (0.37-2.29) | 1.00 | 0.87 (0.35-2.20) | .77 | 74/80 | 0.95 (0.66-1.39) | .85 | 0.96 (0.65-1.42) | .83 |
| rs2593813 | TT | 33/93 | 1[Reference] |  | 1[Reference] |  | 281/362 | 1[Reference] |  | 1[Reference] |  |
|  | TC | 22/76 | 0.82 (0.44-1.51) | .54 | 0.94 (0.49-1.78) | .84 | 265/293 | 1.17 (0.93-1.46) | .20 | 1.35 (1.06-1.72) | **.02** |
|  | CC | 4/10 | 1.13 (0.33-3.84) | 1.00 | 1.38 (0.42-4.58) | .60 | 26/47 | 0.71 (0.43-1.18) | .21 | 0.80 (0.47-1.34) | .39 |
| rs4918 | CC | 36/111 | 1[Reference] |  | 1[Reference] |  | 302/430 | 1[Reference] |  | 1[Reference] |  |
|  | CG | 27/76 | 1.10 (0.61-1.95) | .77 | 1.21 (0.66-2.21) | .54 | 245/293 | 1.19 (0.95-1.49) | .14 | 1.30 (1.01-1.65) | .04 |
|  | GG | 4/5 | 2.47 (0.63-9.68) | .24 | 1.99 (0.48-8.25) | .35 | 33/62 | 0.76 (0.48-1.19) | .27 | 1.33 (0.81-2.18) | .26 |
| ***CYP4F3*** | | | | | | | | | | | |
| rs3794987 | AA | 35/120 | 1[Reference] |  | 1[Reference] |  | 201/200 | 1[Reference] |  | 1[Reference] |  |
|  | AG | 26/59 | 1.51 (0.83-2.74) | .21 | 1.93 (1.02-3.65) | **.04** | 98/132 | 0.74 (0.53-1.02) | .08 | 1.42 (0.97-2.08) | .08 |
|  | GG | 6/7 | 2.94 (0.93-9.31) | .09 | 2.12 (0.68-6.58) | .19 | 11/7 | 1.56 (0.59-4.11) | .47 | 0.64 (0.24-1.69) | .37 |
| rs1159776 | AA | 42/126 | 1[Reference] |  | 1[Reference] |  | 432/518 | 1[Reference] |  | 1[Reference] |  |
|  | AG | 18/62 | 0.87 (0.46-1.64) | .75 | 1.01 (0.52-1.97) | .98 | 156/232 | 0.81 (0.63-1.02) | .08 | 0.81 (0.97-1.03) | .09 |
|  | GG | 2/4 | 1.50 (0.27-8.49) | .64 | 1.62 (0.29-9.08) | .58 | 18/18 | 1.20 (0.62-2.33) | .61 | 1.24 (0.63-2.41) | .53 |
| rs4646519 | TT | 23/68 | 1[Reference] |  | 1[Reference] |  | 142/129 | 1[Reference] |  | 1[Reference] |  |
|  | TC | 33/83 | 1.18 (0.63-2.19) | .64 | 0.96 (0.50-1.85) | .90 | 143/146 | 0.89 (0.64-1.24) | .50 | 0.91 (0.65-1.28) | .59 |
|  | CC | 4/29 | 0.41 (0.13-1.28) | .14 | 0.18 (0.05-0.70) | **.01** | 41/43 | 0.87 (0.53-1.41) | .62 | 0.91 (0.56-1.50) | .72 |
| rs4343407 | CC | 36/88 | 1[Reference] |  | 1[Reference] |  | 137/198 | 1[Reference] |  | 1[Reference] |  |
|  | CT | 31/104 | 0.73 (0.42-1.27) | .32 | 0.86 (0.48-1.55) | .62 | 132/220 | 0.87 (0.64-1.18) | .39 | 0.89 (0.65-1.22) | .46 |
|  | TT | 0/0 | NA | NA | NA | NA | 0/0 | NA | NA | NA | NA |
| rs2683038 | GG | 46/119 | 1[Reference] |  | 1[Reference] |  | 183/273 | 1[Reference] |  | 1[Reference] |  |
|  | CG | 17/62 | 0.71 (0.38-1.34) | .35 | 0.54 (0.28-1.07) | .08 | 65/121 | 0.80 (0.56-1.14) | .25 | 0.73 (0.50-1.07) | .11 |
|  | CC | 4/11 | 0.94 (0.29-3.10) | 1.00 | 0.69 (0.19-2.45) | .56 | 10/17 | 0.88 (0.39-1.96) | .84 | 0.79 (0.34-1.81) | .57 |

Abbreviation: CI, confidence interval; OR, odds ratio; SNP, nucleotide polymorphism. All OR and P value are of the reference group but against the other categories.

a Values adjusted for age, sex.
