# Supplementary material for: Genetic Variation of the Human α-2-Heremans-Schmid Glycoprotein (AHSG) Gene Associated with the Risk of SARS-CoV Infection
Source: PLoS One. 2011 Aug 17;6(8):e23730. doi: 10.1371/journal.pone.0023730 (PMC3163911; doi:10.1371/journal.pone.0023730)
Supplement: Table S3 — Combinatorial Analysis Under the Dominant Model. (DOC) [file pone.0023730.s003.doc]

Table S3 Combinational Analysis Under Dominant Model

|  | | | | | | | |
| --- | --- | --- | --- | --- | --- | --- | --- |
| **SNP** | **Genotype** | **No. of Cases (%)** | **No. of Controls (%)** | **Crude OR (95% CI)** | ***P* Value** | **Adjusted OR (95% CI)^a^** | ***P* Value** |
| ***AHSG*** | | | | | | | |
| rs2248690 | AA | 409 (60.86) | 690 (72.48) | 1[Reference] |  | 1[Reference] |  |
|  | TT/AT | 263 (39.14) | 262 (27.52) | 1.69 (1.37-2.09) | **<.001** | 1.70 (1.37-2.09) | **<.001** |
| rs4917 | CC | 327 (47.67) | 489 (54.21) | 1[Reference] |  | 1[Reference] |  |
|  | TT/CT | 359 (52.33) | 413 (45.79) | 1.30 (1.07-1.59) | **.01** | 1.22 (1.02-1.54) | .08 |
| rs2077119 | CC | 218 (35.39) | 280 (37.58) | 1[Reference] |  | 1[Reference] |  |
|  | AA/AC | 398 (64.61) | 465 (62.42) | 1.10 (0.88-1.37) | .43 | 1.15 (0.92-1.44) | .22 |
| rs2593813 | TT | 314 (49.76) | 455 (51.65) | 1[Reference] |  | 1[Reference] |  |
|  | CC/CT | 317 (50.24) | 426 (48.35) | 1.08 (0.88 -1.32) | .50 | 1.15 (0.93-1.42) | .19 |
| rs4918 | CC | 338 (52.24) | 541 (55.37) | 1[Reference] |  | 1[Reference] |  |
|  | GG/CG | 309 (47.76) | 436 (44.63) | 1.13 (0.93-1.38) | .22 | 1.15 (0.94-1.42) | .18 |
| ***CYP4F3*** | | | | | | | |
| rs3794987 | AA | 236 (62.60) | 320 (60.95) | 1[Reference] |  | 1[Reference] |  |
|  | GG/AG | 141 (37.40) | 205 (39.05) | 0.93 (0.71-1.22) | .63 | 0.85 (0.64-1.14) | .28 |
| rs1159776 | AA | 474 (70.96) | 644 (67.08) | 1[Reference] |  | 1[Reference] |  |
|  | GG/AG | 194 (29.04) | 316 (32.92) | 0.83 (0.67-1.03) | .10 | 0.84 (0.67-1.04) | .11 |
| rs4646519 | TT | 165 (42.75) | 197 (39.56) | 1[Reference] |  | 1[Reference] |  |
|  | CC/CT | 221 (57.25) | 301 (60.44) | 0.88 (0.67-1.15) | .37 | 0.90 (0.68-1.18) | .43 |
| rs4343407 | CC | 173 (51.49) | 286 (46.89) | 1[Reference] |  | 1[Reference] |  |
|  | TT/CT | 163 (48.51) | 324 (53.11) | 0.83 (0.64-1.09) | .20 | 0.83 (0.64-1.09) | .18 |
| rs2683038 | GG | 229 (70.46) | 392 (65.01) | 1[Reference] |  | 1[Reference] |  |
|  | CC/CG | 96 (29.54) | 211 (34.99) | 0.78 (0.58-1.04) | .09 | 0.75 (0.56-1.01) | .06 |
|  | | | | | | | |

Abbreviation: CI, confidence interval; OR, odds ratio; SNP, nucleotide polymorphism. All OR and P value are of the reference group but against the other category.

a Values adjusted for age, sex.
